# Anorectal Cancer with Bone Marrow and Leptomeningeal Metastases

**DOI:** 10.1155/2018/9246139

**Published:** 2018-12-30

**Authors:** Ahmed Zeeneldin, Nasser Al-Dhaibani, Yasser M. Saleh, Amal Mostafa Ismail, Zuhair Alzibair, Mohamed Shafi Moona, Wael Mohamed

**Affiliations:** ^1^Oncology Center, King Abdulla Medical City, Saudi Arabia; ^2^National Cancer Institute, Cairo University, Egypt; ^3^Department of Clinical Oncology and Nuclear Medicine, Mansoura University, Egypt; ^4^Faculty of Medicine, Port Said University, Egypt; ^5^Oncology Center, Saudi German Hospital, Saudi Arabia

## Abstract

This is an interesting case of anorectal signet ring carcinoma with first presentation of an early stage disease, showing the aggressive disease and the undetectable behavior of this type of histology which can mislead diagnosis. Brain/CNS metastasis from colorectal cancer (CRC) is rare occurring in 3% of cases, and leptomeningeal carcinomatosis (LMC) is extremely rare in CRC (<0.02%). Symptoms and signs of LMC are pleomorphic and may be localized to three compartments: cerebral hemispheres, cranial nerves, and spinal cords and roots. Treatment of metastatic rectal cancer has been improving over the last few years with a lot of changes toward longer survival and improvement in quality of life and to change the disease into a chronic condition. However, in our case, the overall survival from the onset of LMC was 3 weeks only. Revising the evidence in the treatment of signet ring histology of rectal cancer, there is no specific treatment recommendation that is for this histology and for such very aggressive behavior which could be considered as a separate entity to the classic adenocarcinoma histology.

## 1. Case Presentation

### 1.1. Clinical Data

This is a case of a 42-year-old male with no significant past medical history. In Feb 2015, he presented with increasing constipation that was later associated with rectal bleeding. In April, there were generalized bone pains involving the chest wall, dorsal spine, and pelvis. PR examination showed a mass almost occluding the lumen located 4 cm from the anal verge.

### 1.2. Lab

His baseline blood count results are as follows: white blood cells 11.6K/ml, neutrophils 8.4K/ml, hemoglobin (Hb) 12.6 g/dl, MCV 85 fl, MCH 30 pg, and platelets 202K/ml. His renal and liver function tests were within normal ranges. His baseline carcinoembryonic antigen was 309 ng/dl.

Proctocolonoscopy showed a mass 4 cm from the anal canal extending proximally for 10 cm. When biopsied, histopathologic examination result confirmed a poorly differentiated adenocarcinoma with focal signet ring morphology arranged in sheets and cords with minimal acinar formation.

### 1.3. Imaging

**Figure 1:**
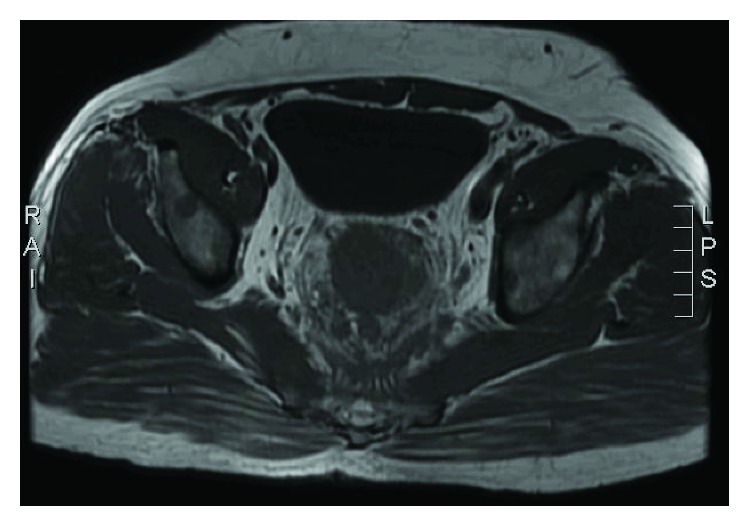
MRI showing the rectal lesion with the perirectal fat stranding.

**Figure 2:**
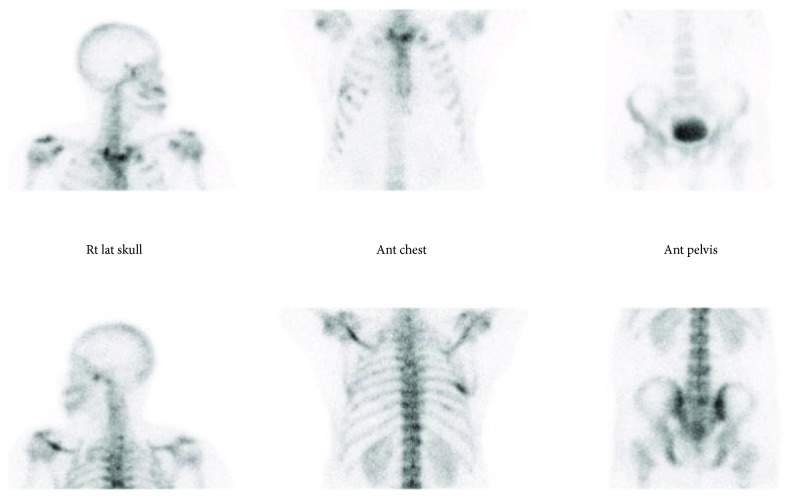
Bone scan showing the widespread osseous deposits.

**Figure 3:**
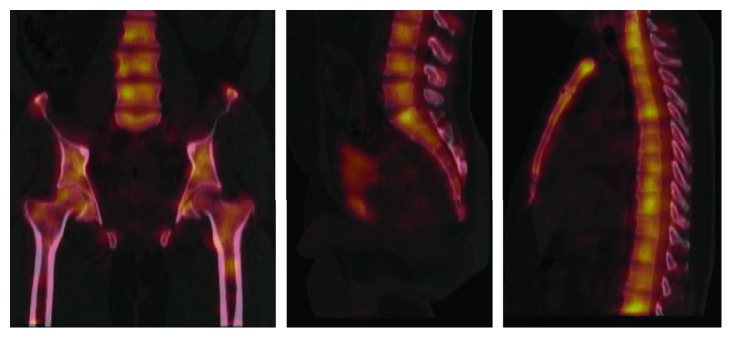
SPECT-CT showing diffuse bone and bone marrow lesions.

Pelvic MRI showed a rectal mass with circumferential thickening and stranding extending to the pelvic side wall (Figure 1) with multiple large left iliac and mesorectal lymph nodes reaching a size of 2.8 cm. Multiple foci of T2 hyperintensity and enhancement were detected involving the lumbar and sacral spine, both iliac bones, and proximal femura. Computerized tomography of chest abdomen and pelvis showed a rectal mass (9 × 5.6 × 5 cm), left iliac lymph node (1.3 cm), a right upper lung nodule (0.3 cm), and no liver metastases. Bone scan confirmed the widespread active osseous lesions involving the spine, ribs, and pelvic bones (Figure 2). SPECT-CT suggested diffuse bone infiltration (Figure 3).

### 1.4. Treatments and Course

**Figure 4:**
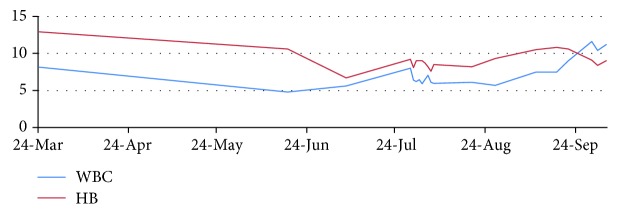
Graph showing the hemoglobin (g/dl) and white cell count (K/ml) of the patient during the disease course.

**Figure 5:**
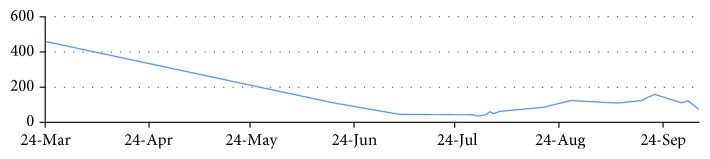
Graph showing the platelet count (K/ml) of the patient during the disease course.

Between June 1 and 14, he received palliative radiotherapy to the pelvis in a dose of 30 grays over 10 fractions with improvement of the rectal bleeding and was scheduled to start palliative chemotherapy, but the patient became pale and developed progressive and prolonged drop in his hemoglobin and platelet levels (Figures 4 and 5). He received multiple units of packed red blood cell transfusions with no improvements in the hematologic parameters. Bone marrow biopsy confirmed the diagnosis of malignant bone marrow infiltration by signet ring cells positive for Ck20 and negative for Ck7 with markedly reduced hematopoietic cells.

The patient had the height of 159 cm, weight of 69 kg, and surface area of 1.75. On July 29th, he started zoledronic acid (4 mg IV) and capecitabine (1500 mg orally twice daily ×14 days) (XELOX protocol) as his condition was not fit to receive FOLFORINOX. He required occasional packed RBC and platelet transfusions during the first few days of capecitabine. Gradually, he started to show clinical improvement in the pains and rectal bleeding as well as in the hematological parameters. At the due time of cycle two on 19/8/2015, his Hb was 8.2 g/dl, platelet was 85K/ml, WBC was 6.2K/ml, and neutrophils were 3.4K/ml. Oxaliplatin in half its usual dose (i.e., 106 mg instead of 212 mg) was added to capecitabine. Zoledronic acid was also given. Anti-EGFR therapy was not added as RAS testing was not yet reported. Bevacizumab was not considered due to thrombocytopenia and the increased risk of bleeding.

At the due time of next cycle on 10/9/2015, his HB was 10.5 g/dl, platelets were 109K/ml, WBC was 6.2K/ml, and neutrophils were 4.1K/ml. At that time, he had oral mucositis grade 2 with dysphagia. Supportive treatment was given, and treatment was hold. On 21/9/2015, his Hb was 10.6 g/dl, platelet was 159K/ml, WBC was 9K/ml, and neutrophils were 7.6K/ml. He showed some improvements of the mucositis and dysphagia. The third cycle was given exactly as the second one (i.e., 50% dose reduction of oxaliplatin in addition to capecitabine and zoledronic acid).

**Figure 6:**
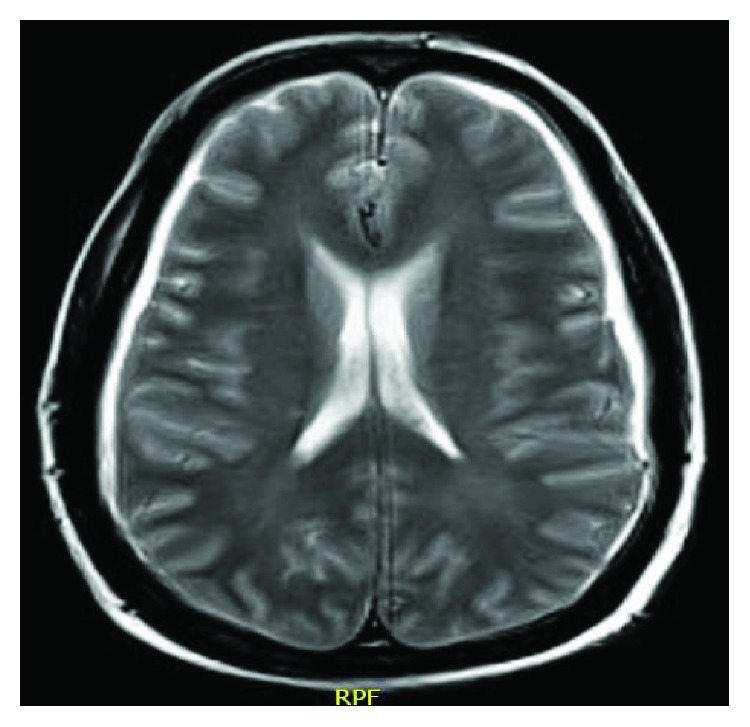
MRI showing leptomeningeal thickening.

**Figure 7:**
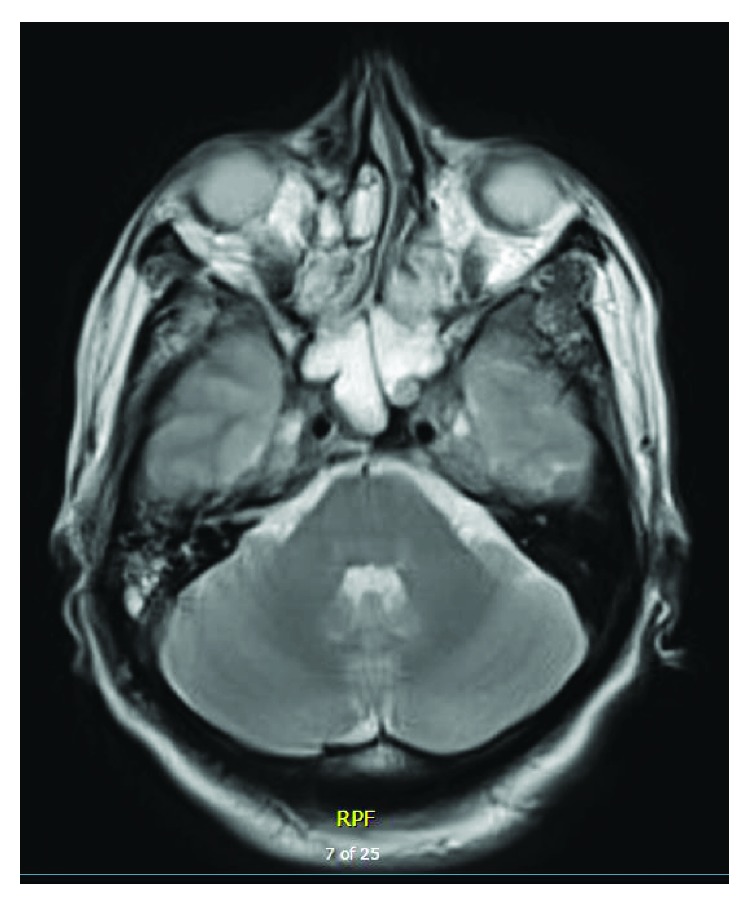
MRI showing sphenoidal and ethmoidal mucosal thickening.

On 29/9/2015, he presented to the Emergency Department with complained of diplopia and nasal regurgitation. His vitals were stable. He had left divergent squint. CT brain showed prominent bilateral hypodense collection more to the left with some linear opacities and diffuse cortical effacement (Figure 6). MRI brain showed diffuse meningeal thickening and enhancement with evidence of sphenoidal and ethmoidal mucosal thickening (Figure 7). His carcinoembryonic antigen tripled to 1049 ng/ml. Radiation oncologists did not encourage the start RT for the fear of an infectious process. CSF was not tapped. Empirical antimicrobials were started including antivirals (acyclovir 500 mg IV q 8 hours ×14 days and oseltamivir 75 mg orally twice daily for 8 days) and antibiotics (ceftriaxone 2000 mg every 12 hours for 15 days, levofloxacin 750 mg intravenously every 24 hours ×5 days, vancomycin 1250 mg intravenously every 12 hours ×7 days). All microbiological tests were negative including aerobic and anaerobic blood cultures, sputum acid fast bacilli, H1N1 and MERS coronavirus screening, Aspergillus galactomannan antigen (0.08), serum (s) blastomyces antibody (ab), Brucella abortus and melitensis tests, s. cocci CF and ID, Coxiella burnetii, cryptococcal antigen (ag), serum histoplasma ab, and Syphilis TP. Patient did not show signs of improvements. Later, high-resolution CT chest showed two right lung nodules and the largest is 0.5 cm in diameter. CT abdomen and pelvis showed progression of rectal lesion and newly developed innumerable small hypodense liver lesions of 0.7 cm diameter. The patient ran a grave course and expired on 17/10/2015.

## 2. Discussion

Worldwide in the year 2012, colorectal cancer (CRC) is the third most common cancer in males and the second in females. It is the fourth cause of cancer death in males and the third in females. In Arab countries, CRC is the first common cancer in males and the second in females. It is the second cause of cancer mortality both in males and females [1, 2].

Histologically, the WHO classified colorectal carcinoma into adenocarcinoma (AC), medullary carcinoma, mucinous AC, signet ring cell carcinoma (SRCC), and some other subtypes. In mucinous AC (MAC), >50% of the lesion is extracellular mucin, and in SRCC, >50% of the tumor is signet ring cells with prominent intracytoplasmic mucin [3]. Almost 96% of SRCC originate in the stomach and the rest in the colon, rectum, pancreas, urinary bladder, and breast [4]. SRCC constitutes 1.1% of colorectal cancers [5].

Colorectal SRCCs are present at an advanced stage with T3 or T4, positive LN, and distant metastases. They have poor survival [5, 6]. They are prognostically unfavorable independent of the stage [3]. Compared with AC, colorectal SRCC tends to occur at younger age, to affect the right side, to be poorly differentiated, to recur more, and to have poor survival [5, 6]. They express mucin-related genes (HATH1, MUC2, and SOX2) and show disruption of the E-cadherin/beta-catenin complex, amplification of Bcl-2 [6], low K-ras, high BRAF mutations, and high MSI [7]. Loss of protein expression of E-cadherin may contribute to the high-grade and invasive nature of colorectal SRCC [7]. In the current case, the patient was young (42 years) and had anal/rectal lesion that is locally advanced (T3) associated with distant metastases. He ran a rapid grave course.

Skeletal metastases (SKM) in CRC may reach up to 24% if radiotherapy or systemic therapies are not used [8] and to 5.5–11% when these therapies are used [9–11]. Isolated SKM is rare, occurring in 1–2% of CRC [8]. It is usually associated with liver or lung metastases [8, 9]. SKM is more in younger patients, in rectum and caecum primarily, and in SRCC [8–10]. CRC SKM affects the vertebral column in 65%, hip/pelvis in 34%, long bones in 26%, and other bones in 17% [10]. SKM usually presents with bone pains or pathological fractures [12]. Time from CRC diagnosis to onset of SKM ranges from 10 to >5000 days with a median of 11 months [10]. Skeletal scintigraphy detects SKM with high sensitivity. Palin X-rays are helpful in symptomatic lesions and in assessing fracture risk or questionable scintigraphic findings. CT is helpful when other imaging techniques are unclear. Whole-body MRI and PET-CT are the most sensitive and specific methods for the detection of SKM. Hybrid techniques like SPECT-CT, PET-CT, and PET-MRI combine the strengths of their different components and avoid the weaknesses [13]. Traditional treatments for SKM include radiation, surgery, and chemotherapy [14]. Both denosumab and zoledronic acid reduce pains and skeletal morbidities in patients with SKM [12, 15]. In 5 years, 38% of patients isolated SKM are alive compared to 16% when other metastases coexist [11]. The median survival is 5–7 months [10, 16]. In the current case, SKM was the initial site of metastasis and developed 4 months following initial diagnosis. It presented with bone pains and tenderness and confirmed by bone scintigraphy and SPECT/CT. He received palliative RT, zoledronic acid, and systemic chemotherapy with mild improvement. The overall survival from the onset of SKM was 5 months.

Bone marrow metastasis (BMM) was noticed at necropsy in 93/1541 CRC patients (11.6%) where chemotherapy was rarely used. It is extremely rare (<1%) in the absence of liver and lung metastases and increases to 3% with both liver and lung metastases [17]. The most common metastatic site associated with BMM from solid tumors is the bone in 70% of cases followed by liver (14%), brain (10%), and lung (10%) metastases [18]. Early features of BMM include microangiopathic hemolytic anemia and leukoerythroblastosis in 70% of cases. Clinically, patients present with anemia in 68%, thrombocytopenia in 58%, and neutropenia in 23%. Bicytopenia is encountered in 32% and pancytopenia in 18%. About 15% of cases had no hematological findings and were suspected based on imaging only [18, 19]. Using modern techniques, malignant cells were found in BM in 21% of patients with stages I–III at the time of curative surgery [20]. However, the clinical relevance of micrometastasis in BM is to be validated. Mostly, its value may be limited unless supported by some other clinical findings, such as a leukoerythroblastic reaction or abnormal peripheral blood counts [21]. Despite BM biopsy is the surest and fastest method of diagnosis, MRI and FDG PET or PET/CT are very sensitive in the detection of BMM [21, 22].

BMM from CRC is primarily treated by chemotherapy with or without targeted therapy. Agents most commonly used are fluoropyrimidines (5-FU, S1) and oxaliplatin and occasionally bevacizumab. These lead to clinical improvements in many patients [23–28]. Cytopenias secondary to BMM not only increase the risk of infection and bleeding but also hinder the delivery of effective chemotherapy [18]. BMM usually carries a poor prognosis with a median survival of 5–7 months [29]. Performance status, other metastatic sites, and platelet counts are important prognosticators. Survival of patients with thrombocytopenia is shorter than with normal platelet counts (1 month vs. 13 months) [18].

Brain/CNS metastasis from CRC is rare occurring in 3% of cases [10]. Leptomeningeal carcinomatosis (LMC) is extremely rare in CRC (<0.02%) [30]. It is rarely the sole site of intracranial disease [31]. Symptoms and signs of LMC are pleomorphic and may be localized to three compartments: cerebral hemispheres, cranial nerves, and spinal cords and roots. Headache, diplopia, and back/neck pain are common. CSF cytology is the gold standard in LMC diagnosis. Although CSF cytology has 75% sensitivity and 100% specificity, it has a low negative predictive value. CT scans are abnormal in 25–56% of cases with LMC. However, contrast-enhanced MRI has higher sensitivity (59%) and specificity (95%) [32]. Without treatment, the median survival of patients with LMC is 4–8 weeks. Patients usually die of progressive leptomeningeal or systemic cancer [30, 32, 33]. Bulky leptomeningeal disease, concurrent CNS disease, epidural cord compression, and poor performance status are poor prognostic factors [32]. Intrathecal or systemic chemotherapy may be used with limited efficacy [34]. In the current case, LMC was suspected clinically based on the neurological findings (diplopia and squint) and MRI. However, the concomitant mucosal thickening of the paranasal sinus raised the issue of secondary meningitis. However, the lack of response to a multitude of antimicrobials was going with LMC. The overall survival from the onset of LMC was 3 weeks.
